# Shallow Failure of Weak Slopes in Bayan Obo West Mine

**DOI:** 10.3390/ijerph19159755

**Published:** 2022-08-08

**Authors:** Wencai Wang, Yongfu Yan, Yue Qu, Pengfei Wang

**Affiliations:** 1 College of Mining and Coal, Inner Mongolia University of Science and Technology, Baotou 014010, China; 2 Baogang United Steel Barun Company, Baotou 014010, China; 3 Inner Mongolian Zhengchuang Technology Co., Ltd., Ordos 017000, China; 4 College of Mining Engineering, Taiyuan University of Technology, Taiyuan 030024, China

**Keywords:** weak rock slope, shallow slide, strength weakening, pre-split blasting, creep-pull crack

## Abstract

The slope stability of large open-pit mines has always been a concern and the analysis of large-scale slope landslides is a focus. However, shallow failure in soft rock slopes also has a serious impact on safe production. The northern slope of Baiyunebo West Mine has many shallow landslides in the final slope, resulting in damage of the maintenance channel of the belt transportation system, which has a serious impact on the safety of production. In order to reduce the shallow failure in weak rock slope, it is necessary to analyze the behavior and characteristics of shallow failure in weak rock. Firstly, the mechanical parameters of the intact rock were obtained by using the exploration data; secondly, through the analysis of blasting-damage range, the distribution characteristics of fractures after the failure of weak rock were obtained. Finally, through theoretical analysis, numerical simulation, surface displacement monitoring and on-site shallow-failure case analysis, the deformation and characteristics of shallow failure of weak rock slope in West Mine were obtained. It was found that the mechanical parameters of rock mass strength on the surface of weak rock slope and the original rock were quite different after mining disturbance. The mode of failure of shallow weak rock slope in the West Mine was creep-cracking; the numerical modelling analysis was carried out by using the assignment method of shallow lithology weakening and gradual change, which is more in line with the deformation characteristics of weak rock slope in West Mine. The lower deformation of the soft rock slope in West Mine is 3–5 times that of the upper deformation. The research results are helpful to understand the influence of blasting on the stability of soft rock slope. At present, West Mine has started to adjust blasting parameters according to the research results, so as to reduce the excessive damage of blasting to rock mass, so the stability of the slope is improved.

## 1. Introduction

The Bayan Obo West Mine is a large-scale, open-pit metal mine located in the central and western region of Inner Mongolia. At present, the stope depth is more than 200 m. The rock mass composition of the slope is relatively complex. The upper part is dominated by tertiary and weathered slate, the middle part is fragmented slate, and the middle and upper parts are weak rock slope. At present, the main transportation channel of the north side of the stope and the maintenance channel of the belt transportation system are all distributed in the weak rock slope. Collapse will have a serious impact on production of the mine.

A study by Yang [1] and Zhou [2] showed that disturbed weak rock slopes are prone to collapse when encountering water, and the water in the slope increases the weight of the slope and reduces the anti-sliding force. The study by Song [3] showed that rainfall and water catchment cause the creep of weak rock slopes, which makes the slope change from the original stable state to a creep state. The study by Bao [4] showed that creep does not occur in the deep rock mass, but only in the shallow area. The study by Lou [5] showed that for the slope with weakened rock mass, numerical simulation analysis can be carried out by reducing the mechanical parameters when calculating its stability. The study by Luo [6] showed that vibration can induce instability in slopes, especially weak rock slopes. The study by Zhan [7] showed that the failure of soft rock slope is generally progressive, and it is generally a progressive failure mode from the slope toe to the inside of the slope.

**Figure 1:**
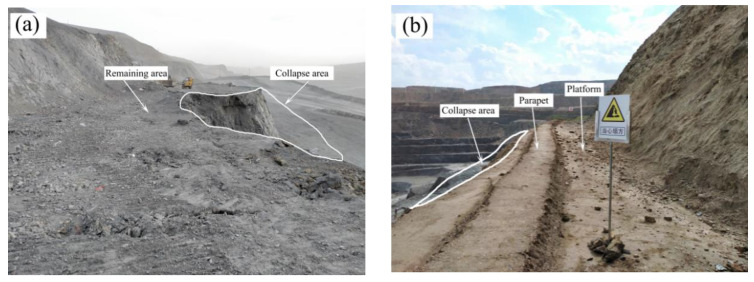
Shallow damage bench: (**a**) Carbonaceous slate bench collapse; (**b**) Repaired soft rock bench.

For the case of Bayan Obo West Mine, in 2018, boundary optimization was carried out, and the upper slope of the stope was re-supported, so the length of the newly exposed slope exceeded 21 km. These freshly exposed slopes did not show the expected stability. Instead, there have been superficial collapses in many places. These landslides were mainly located in the north side of the stope, and they were basically small-scale shallow landslides with a single step. The thickness of the landslide body was 2–4 m, and the length was 10–30 m, because the width of the current platform on the side of the West Mine is 10 m. The safety retaining wall occupies a platform width of 1.5 m, the slope protection at the foot of the slope occupies 0.5 m, and the narrowest width allowed for passing vehicles is 5 m, so the surplus width of the platform is less than 3 m (Figure 1).

## 2. Analysis on the Deformation Mechanism of the Shallow Layer of Soft Rock Slope

### 2.1. Analysis of Blasting-Damage Layer

For weak rock slopes, after experiencing vibration from blasting, and the cyclic action of bench-blasting vibration [8,9,10] and long-term natural weathering [11], then there will be a fractured layer with gradually decreasing new cracks from the surface to the inside near the free surface of the slope [12]. Rock masses with different depths have different fragmentation degrees, and different rock mass fragmentation states have different rock mass mechanical properties. Therefore, when analyzing shallow failures, the deformation characteristics of the fragmentation model at different depths in the shallow layer of the fractured weak rock slope should be considered. At present, the main support technology used in the West Mine is the pre-split controlled-blasting technology, which has obvious effects on protecting the slope rock mass from excessive blasting vibration damage [13].

(1) Fracture extension range

Slope blasting uses pre-split controlled blasting; it can protect the retained rock mass to a certain degree, but the rock mass will still be damaged to a certain extent [14]. According to the effect of explosives, there is a crushed zone, a fractured zone and a vibrated zone in order from the blast hole outward. The crushed zone is the fragmentated area produced by the compression of the explosive. The fractured zone is the area produced by the action of the stress wave of the explosive, and the vibrated area is generally regarded as the elastic action area [15,16,17]. It is generally believed that the best pre-splitting control blasting is when the crushing zone just extends to the blast hole wall, uses very little explosives, and reduces the damage to the rock mass [18]. The charge structure of pre-split controlled blasting is uncoupled charge, and there are many empirical formulas for the selection of charge structure [19]. However, Dai’s [20] research and calculation formula on crushed circle and fractured circle during blasting can more comprehensively reflect the feedback of rock to blasting.

The formula for calculating the radius of the crushed circle in the state of uncoupled charge is as follows:

(1) $$R_{1}={\left(\frac{\rho_{0}D^{2}nk^{-2\gamma }l_{e}B}{8\sqrt{2}\sigma_{\mathrm{cd}}}\right)}^{\frac{1}{\alpha }}r_{b}$$

The formula for calculating the radius of the fractured circle in the uncoupled state is as follows:

(2) $$R_{2}={\left(\frac{\sigma_{R}B}{\sqrt{2}\sigma_{\mathrm{td}}}\right)}^{\frac{1}{\beta }}R_{1}$$

(3) $$B={\left[{\left(1+b\right)}^{2}+\left(1+b^{2}\right)-2\mu_{d}\left(1-\mu_{d}\right){\left(1-b\right)}^{2}\right]}^{\frac{1}{2}}$$

where *R*_1_ is the blasting crushed-circle radius; *R*_2_ is the radius of the blasting fractured circle; *ρ*_0_ is the density of the explosive; *D* is the detonation velocity of the explosive; *α* is the decay index of the explosion load during the propagation process; *β* is the decay index of the explosion load during the propagation process outside the crushed circle; *σ*_cd_ is the dynamic compressive strength of the rock; *σ_td_* is the dynamic tensile strength of the rock; *r_b_* is the blast hole radius; *b* is the lateral stress coefficient; *γ* is the expansion adiabatic index of the detonation product; *n* is the pressure increase coefficient when the explosive collides with the wall of the blast hole, which is generally 10; *k* is the radial uncoupling coefficient of the charge; *le* is the charge axial coefficient; *μ_d_* is the dynamic Poisson’s ratio of the rock.

In order to control the range of the crushing circle, the optimal expectation is when the radius of the crushing circle is equal to the radius of the blast hole, and the crack circles just penetrate each other, so that a flat pre-split surface can be formed and the integrity of the original rock can be maintained to the greatest extent. Therefore, *R*_1_ is generally considered to be the radius of the pre-split hole, and the radius of the crack circle *R*_2_ during pre-split blasting can be obtained by Equation (2).

(2) Parameter modification

In the implementation of pre-splitting blasting in West Mine, due to the convenience of the construction operation, the pre-splitting explosive column was naturally lowered into the pre-splitting hole directly along the pre-splitting hole wall without using the locator. This leads to the pre-splitting charge column not being located at the center of the blast hole, but at the eccentric and uncoupled charge mode close to the side of the rock mass. In this case, the crack circle radius *R*_2_^′^ of pre-splitting blasting is no longer applicable to Equation (2), so it is necessary to modify the calculation formula. According to the research results [21], there is a certain proportional relationship between the fractured zone of eccentrically uncoupled and coupled charges.

(4) $${R_{2}}^{\prime }=\lambda \cdot {\left(\frac{\sigma_{R}B}{\sqrt{2}\sigma_{td}}\right)}^{\frac{1}{\beta }}\cdot R_{1}$$

where the coefficient *λ* is the correction coefficient, which means the ratio of the fractured zone between the eccentric coupling charge and the concentric decoupling charge under the same working condition. According to the research results of Yang [21], the relative size of the damage variable of the uncoupled eccentric charge structure in the fractured zone satisfies: eccentric coupling side > concentric uncoupling > eccentric uncoupling. Additionally, according to the ratio relationship obtained by the laboratory simulation, it can be considered that *λ* = 1.73, and, thus, ${R_{2}}^{\prime }=1.73\cdot {\left(\frac{\sigma_{R}B}{\sqrt{2}\sigma_{td}}\right)}^{\frac{1}{\beta }}\cdot R_{1}$.

According to Zhang’s [22] results of borehole TV measurement after pre-split blasting on the rock slope, as the distance is far from the blasting source, the degree of development of joints and fractures in the rock mass gradually decreases, and the disturbed depth of the rock mass is 9 m; beyond this distance, the rock mass maintains the state of primary joints. According to Wang’s [23] research on coal seam pre-split blasting, when the diameter of the blast hole is 80 mm, the diameter of the grain column is 64 mm, the distance between the blast holes is 6 m, the uncoupling coefficient is 1.25, and the maximum fractured circle radius is 3.5 m. According to the summary and analysis by Huang [24], the current theoretical analysis generally believes that the fractured circle of uncoupled charge blasting is 10–15 *r_b_*, and with the increase in the uncoupling coefficient, the ratio of the fracture radius of the rock mass to the radius of the blasthole presents that there is a decreasing trend from fast to slow. However, there are also studies that believe the actual impact range of blasting is larger; according to the research by Dai [25], it is believed that the impact range of blasting cracks can reach 120–150 *r_b_*. In the fractured circle damaged by blasting, since the integrity of the rock mass has changed, it can be considered that the mechanical properties of the rock mass have also changed. For the accuracy of the study, the rock mass mechanical parameters are accurately calculated and selected within the blasting-damage range.

### 2.2. Determination of Key Mechanical Parameters of Rock Mass

In order to study the strength of the rock mass, Hoke proposed a *GIS* geological-strength-index classification system in 1994 [26], which has been modified and improved many times in the later period, and the system has been applied by most of the geotechnical engineering industry. In 2013, Hoke proposed the relationship between *GIS* and joint state *JCond*_89_ and rock quality index *RQD*:

(5) $$GIS=1.5JCond_{89}+RQD/2$$

The rock mass of the slope is damaged and eroded by blasting, and the final slope rock mass and the original rock will have a certain change in geological strength, which will lead to a certain difference in the stability of the slope rock mass. In Hoke’s geological-strength-index classification system, *JCond*_89_ can be comprehensively evaluated and selected according to joint trace length, fracture width, roughness, filling type and degree of weathering. The rock mass geological strength of the core extracted by geological exploration can be considered as the embodiment of the original rock strength, and the *JCond*_89*core*_ is used to represent the joint state of the original rock. After mining, the exposed slope rock mass can be considered as the rock mass strength after being disturbed and damaged, and the *JCond*_89*slope*_ surface is used to represent the exposed rock mass joint state. Among them, the *JCond*_89*core*_ can be obtained by the observation of the core, and the *JCond*_89*slope*_ can be obtained by the observation of the rock mass on the side slope.

*RQD* refers to the rock quality index [27], and the initial *RQD* value describes the integrity of the core obtained [28].

(6) $$RQD=L_{p}/L_{t}$$

where *L*_p_ refers to the sum of the length of a drilled core greater than 10 cm; *L*_t_ refers to the sum of all the drilling footage.

The initial *RQD* value can be obtained from the drilled core for the first time, but the integrity of the core cannot fully reflect the quality of the rock. Chakraborty. A. K’s [29] research concluded:

(7) $$RQD=115-3.3J_{v}$$

where *J*_v_ refers to the number of volume joints; according to the research results of Du [30], the number of volume joints is a three-dimensional quantity, but it can be obtained by converting the number of one-dimensional joints; *J*_v_ = *K*·*λ*, *λ* is the number of structural surfaces per meter; *K* is a coefficient; and, generally, *K* is taken as 2.0. The *RQD* value calculated by the number of volume joints can measure the integrity of the slope rock mass, which makes the evaluation of rock quality more convenient.

The *RQD_core_* is used to represent the rock quality index calculated from the integrity of the core, and the *RQD_slope_* is used to represent the rock quality index calculated from the structural plane.

### 2.3. Calculation of Rock Mechanics Parameters

According to the generalized Hoke–Brown criterion [31],

(8) $$\sigma_{1}=\sigma_{3}+\sigma_{ci}{\left(m_{b}\frac{\sigma_{3}}{\sigma_{ci}}+s\right)}^{a}$$

where *σ_ci_* is the uniaxial compressive strength of rock blocks; *m_b_*, *s*, and *a* are semi-empirical parameters representing rock mass properties, where:

(9) $$m_{b}=m_{i}e^{\left(\frac{GIS-100}{28-14D}\right)}$$

(10) $$s=e^{\left(\frac{GIS-100}{9-3D}\right)}$$

(11) $$a=\frac{1}{2}+\frac{1}{6}\left(e^{-GIS/15}-e^{-20/3}\right)$$

where *m_i_* is the rock material constant, obtained from the complete rock triaxial compression test, or selected according to experience; *D* is the weakening factor of the rock mass disturbed by human factors or natural factors; and for the weak rock mass slope, controlled blasting takes 0.7.

When the Hoke–Brown criterion parameters are obtained, the corresponding equivalent internal friction angle *φ* and equivalent cohesion *c* can be calculated.

(12) $$\varphi =\sin^{-1}\left[\frac{6am_{b}{\left(s+m_{b}\sigma_{3n}\right)}^{a-1}}{2\left(1+a\right)\left(2+a\right)+6am_{b}{\left(s+m_{b}\sigma_{3n}\right)}^{a-1}}\right]$$

(13) $$c=\frac{\sigma_{\mathrm{ci}}\left[\left(1+2a\right)s+\left(1-a\right)m_{b}\sigma_{3n}\right]{\left(s+m_{b}\sigma_{3n}\right)}^{a-1}}{\left(1+a\right)\left(2+a\right)\sqrt{1+\left[6am_{b}{\left(s+m_{b}\sigma_{3n}\right)}^{a-1}\right]/\left[\left(1+a\right)\left(2+a\right)\right]}}$$

where *σ*_3_*_n_* = (*σ*_3__max_/*σ*_*ci*_) *σ*_3__max_ is the upper limit of the minimum principal stress, which is related to the type of rock mass engineering, for the rock mass of slope engineering, it can be determined by the following formula:

(14) $$\frac{\sigma_{3\max}}{\sigma_{\mathrm{cm}}}=0.72{\left(\frac{\sigma_{\mathrm{cm}}}{\gamma H}\right)}^{-0.91}$$

where *H* is the slope height; *γ* is the weight of the rock mass; and *σ*_cm_ is the overall strength of the jointed rock mass, when: *σ_t_* < *σ*_3_ < (*σ*_*ci*_/4)

(15) $$\sigma_{\mathrm{cm}}=\sigma_{\mathrm{ci}}\frac{\left[m_{b}+4s-a\left(m_{b}-8s\right)\right]{\left(m_{b}/4+s\right)}^{a-1}}{2\left(1+a\right)\left(2+a\right)}$$

By substituting the rock mass mechanical parameters of the slope surface and the core into the above formulas, the equivalent internal-friction angles *φ_slope_* and *φ_core_* and the equivalent cohesion forces cslope and ccore of the rock mass of the core and the slope surface can be calculated.

### 2.4. Analysis of Water Content in Slope

**Figure 2:**
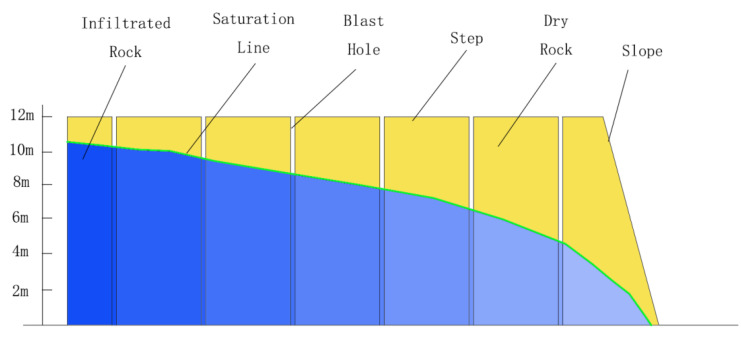
Step waterline position.

The distribution of groundwater in this area can be obtained by measuring the water level of blasting holes. The groundwater level in the north side of the stope shows a continuous distribution trend, and the infiltration line of groundwater increases from the beginning of the step slope to the back. The highest water level is basically 1–2 m away from the top of the slope (Figure 2). Slope collapse is closely related to water [32,33,34]. In the case of water, the slope rock mass cannot remain stable even if the slope angle reaches the natural angle of the repose of the rock. The weakening effect of water on the slope mainly has three aspects: first, under the infiltration of water, the weight of the rock mass will increase, resulting in the increase in sliding force; second, the infiltration of water will reduce the strength of the weak layer and reduce the anti-sliding force; third, water in the rock mass will produce hydrostatic pressure and hydrodynamic pressure, which will also increase the sliding force of the rock mass. When the position of the plane of weakness of the slope-sliding body cannot be clearly defined for the fractured rock mass, in order to study the pertinence of the problem, the effect of water on the rock mass is generally converted into the increase in rock bulk density. In numerical simulation analysis, the infiltration line can also be set in slope stability analysis according to the groundwater level. According to the long-term observation of the water level of the rock mass in the northern slope of the Western Mine, the water level of the bench rock mass fluctuates in a certain range, indicating that the water pressure inside the rock mass is not the same. Due to the differences in geological structure and rock mass structure, the water pressure borne by the slope rock mass is different.

## 3. Slope Stability Analysis

### 3.1. Mechanical Parameters of Shallow Rock Mass

Different rock mechanics parameters will have a serious impact on the stability analysis and calculation of the slope, so in the process of slope stability analysis and calculation, the selection of key rock mechanics parameters is very important. For the shallow failure of the weak rock slope, the ability to accurately describe the lithology of the shallow layer of the slope is the basis for obtaining a good analysis and calculation. According to Equations (1)–(14), by substituting the measured rock mass mechanical parameters and semi-empirical values, the equivalent internal-friction angles *φ_slope_* and *φ_core_* and equivalent cohesion forces cslope and ccore of the rock core and slope face rock mass can be calculated. When the rock mass is damaged by blasting to form a fractured circle, with the attenuation of blasting energy, the size of the crack will decrease, and the effect of crack propagation in the rock under blasting will gradually weaken as it moves away from the blast hole [35,36]. According to the research results of Huang [24], the damage law of blasting rock mass conforms to the inverse “S” curve, and the analysis of the damage change characteristic curve shows that the curvature of the curve in the fracture section changes relatively gently, which can be considered as a linear function. Therefore, it can be considered that the equivalent internal friction angle of the rock mass *φ* is:

(16) $$\varphi =\varphi_{slope}-\frac{\varphi_{slope}-\varphi_{core}}{R_{1}}\cdot L$$

Similarly, the equivalent cohesion *c* of the rock mass *c* can be considered as:

(17) $$c=c_{slope}-\frac{c_{slope}-c_{core}}{R_{1}}\cdot L$$

where: *R*_1_ is the radius of the fracture circle; *L* is the distance between the fractured rock mass and the slope, *L ≤* *D*.

### 3.2. Deformation Analysis of Shallow Rock Mass on Weak Rock Slope

The numerical simulation analysis has a good effect on obtaining the stability analysis and calculation of complex lithological slopes, and can more vividly and intuitively represent the shallow potential deformation of the fractured and weak rock mass. Geo slope [37] is used to analyze the slope stability in the original rock state and after the damage of shallow rock mass and the action of groundwater. When Eldin [38] studied the landslide disaster, he observed that the thickness of the same type of weak rock slope in the field was different due to the different degree of fragmentation of the shallow rock mass. Wu’s [39] research on the on-site reinforcement project of a slope showed that changing the surface rock mass strength of a weak slope can reduce the risk of slope collapse. According to the previous research results, the mechanical parameters of the original rock can be used to assign values in the stability analysis of the original rock steps. However, when analyzing the stability of the side slope after disturbance, it is necessary to assign values to the shallow damaged slope layer by layer. The assignment law is: the mechanical parameters of rock mass in the slope area are assigned according to the measured average, the thickness of rock-mass-weakening layer is based on the action range of blasting vibration fractures, and the remaining rock mass is assigned according to the original rock mechanical parameters.

**Figure 3:**
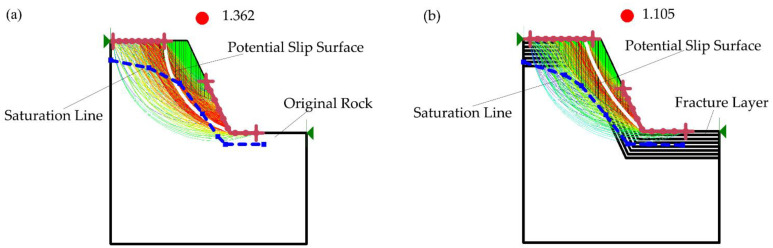
Simulation analysis of shallow damage: (**a**) Stability analysis of original rock slope; (**b**) Stability analysis of failed rock mass slope.

When the mechanical parameters obtained by the core are used for simulation analysis, the obtained slope safety factor is 1.362 (Figure 3a). According to the specification [40], it can be considered that the shallow slope is in a stable state. However, when the gradient mechanical parameter assignment method of strength weakening is used for simulation analysis, the obtained slope safety factor is 1.105 (Figure 3b), and the shallow slope is in a state of insufficient safety reserve. In addition, the thickness of the model damage under the gradual change in rock mechanics is about 0.5 times that under the original parameter model.

### 3.3. Slope Surface Displacement Monitoring

**Figure 4:**
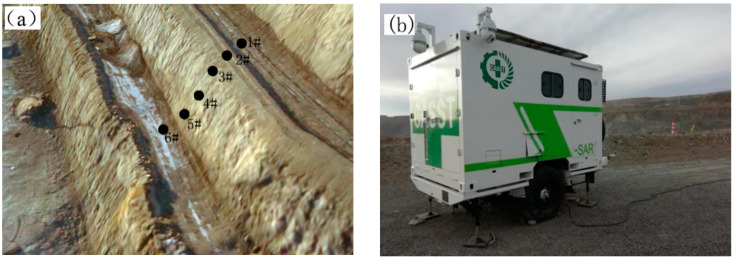

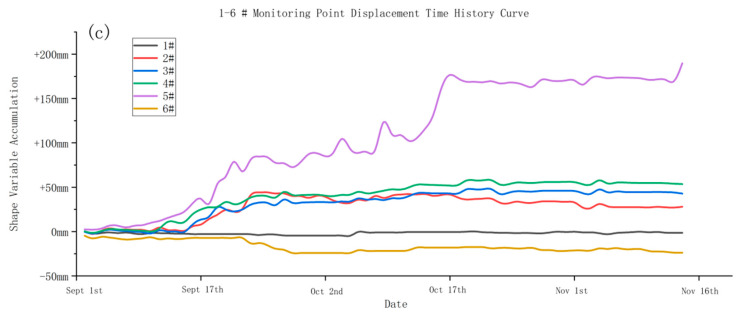
Surface displacement monitoring: (**a**) Displacement-monitoring point; (**b**) Trailer radar; (**c**) Displacement time history curve of monitoring point.

By monitoring the deformation of the slope surface, the deformation of the slope rock mass can be reflected. The West Mine has In-SAR (Figure 4b) technology which has sub-millimeter surface-displacement-monitoring accuracy and can continuously collect the displacement and deformation of the slope surface. By selecting points on the slope surface and different areas of the bench, and analyzing the point displacement, the real deformation of the fragmented soft rock slope can be evaluated.

Through 73 days of continuous data monitoring and statistics on the slope surface, at the position of the slope survey line (Figure 4a): the monitoring point with the largest displacement was the 5# point at the foot of the slope, and its cumulative deformation was 189.66 mm; at point 3# at the top of the slope, its cumulative deformation was 42.8 mm; and the cumulative deformation of point 4# in the middle was 39.76 mm, and the cumulative deformation of other points did not exceed 30 mm (Figure 4c). This shows that the maximum displacement of the weak rock slope in the West Mine occurs at the lower part of the slope, and with the increase in the slope surface, the displacement of the slope gradually decreases, and the displacement at the step bench is the smallest. According to the characteristics of the slope failure mode [41], the deformation law of the shallow damage of the fractured soft rock slope in the West Mine conforms to the creep–pull fracture mode.

### 3.4. Case Analysis of Shallow Failure

A number of local shallow damages occurred in the weak rock slope of the West Mine after the boundary. The damaged body was in a state of fragmentation, and the thickness of the damaged body was less than 3 m. The local collapse of the steps is usually treated by reducing the slope angle and reducing the load and pressing the slope foot, but these two methods will cause width reduction in the upper bench and the lower bench, so the shallow damage will affect the upper and lower steps. In the actual area where the slope collapse occurred, there was local water seepage on the slope surface, the rock mass was relatively broken, and the thickness of the collapsed body was about 3.5 m, which was extremely similar to the results of the simulation analysis.

**Figure 5:**
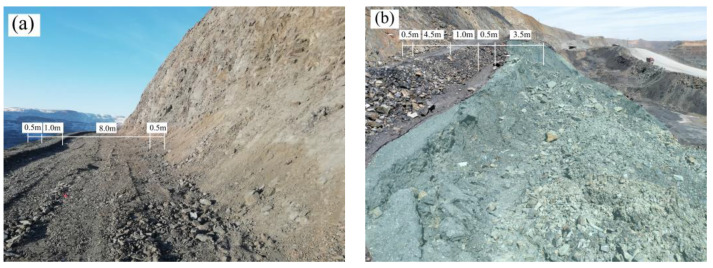
Site collapse comparison: (**a**) Normal bench; (**b**) Post-collapse bench.

The bench width of the northern slope of the West Mine is generally 10 m. The slope toe takes up 0.5 m, the safety-retaining wall takes up 1 m, the shoulder takes up 0.5 m, and the road width is 8 m (Figure 5a). In March 2020, a shallow failure occurred on the side slope of the weak-rock fractured rock mass. The thickness of the damage was about 3.5 m, and the amount of collapsed soil was about 1540 m^3^. The effective road width of the remaining bench was less than 4.5 m (Figure 5b). The damage directly led to the loss of the ability of the bench. The bench width of the remaining bench could only meet the traffic of the slope patrol personnel, resulting in the bench, up to 1.1 km, being unable to be cleaned and maintained by mechanical equipment. The damage at the site started from the local area to drop; then, fractures appeared on the slope surface, and then bulges appeared at the lower part of the slope surface, and finally, failure occurred. The analysis shows that the shallow damage of the weak rock slope conforms to the slope failure mode of creep–fracture. The deformation at the foot of the slope is earlier than that at the top of the slope. The collapsed rock mass is more broken, and the integrity of the rock mass left behind is relatively better.

## 4. Conclusions

(1) After disturbance, the mechanical strength parameters of the shallow rock mass of the weak rock slope in West Mine are obviously inferior to intact rock.
(2) The shallow failure of the weak rock slope in the West Mine experienced such a process where the slope body first underwent large deformation and displacement along the slope toe; then, the slope rock mass began to collapse, and finally, the creep at the bottom of the step pulled the top apart to form a shallow collapse. This deformation and failure mode conforms to the creep–crack mode.
(3) The numerical simulation was carried out by using the parameter-assigning method for weak rock slope layer by layer. The simulation results were very close to the real slope slide failure, which shows the scientificity of this method.
(4) The rule of cumulative displacement and deformation of the soft rock slope of the West Mine is that the toe of the slope is 3–5 times that of the top of the slope.

When studying the deformation of a broken soft-rock slope, water also causes freeze–thaw damage to the slope, so the analysis of cyclic freeze–thaw could be considered when studying this kind of problem in the future.

## Data Availability

Data is available from the authors upon reasonable request.
